# Interconnected growing self-organizing maps for auditory and semantic acquisition modeling

**DOI:** 10.3389/fpsyg.2014.00236

**Published:** 2014-03-20

**Authors:** Mengxue Cao, Aijun Li, Qiang Fang, Emily Kaufmann, Bernd J. Kröger

**Affiliations:** ^1^Laboratory of Phonetics and Speech Science, Institute of Linguistics, Chinese Academy of Social SciencesBeijing, China; ^2^Department of Special Education, Faculty of Human Sciences, University of CologneCologne, Germany; ^3^Neurophonetics Group, Department of Phoniatrics, Pedaudiology, and Communication Disorders, Medical School, RWTH Aachen UniversityAachen, Germany; ^4^Cognitive Computation and Applications Laboratory, School of Computer Science and Technology, Tianjin UniversityTianjin, China

**Keywords:** neural network, neurocomputational models, interconnected growing self-organizing map, auditory feature map, semantic feature map, auditory–semantic association, language acquisition

## Abstract

Based on the incremental nature of knowledge acquisition, in this study we propose a growing self-organizing neural network approach for modeling the acquisition of auditory and semantic categories. We introduce an Interconnected Growing Self-Organizing Maps (I-GSOM) algorithm, which takes associations between auditory information and semantic information into consideration, in this paper. Direct phonetic–semantic association is simulated in order to model the language acquisition in early phases, such as the babbling and imitation stages, in which no phonological representations exist. Based on the I-GSOM algorithm, we conducted experiments using paired acoustic and semantic training data. We use a cyclical reinforcing and reviewing training procedure to model the teaching and learning process between children and their communication partners. A reinforcing-by-link training procedure and a link-forgetting procedure are introduced to model the acquisition of associative relations between auditory and semantic information. Experimental results indicate that (1) I-GSOM has good ability to learn auditory and semantic categories presented within the training data; (2) clear auditory and semantic boundaries can be found in the network representation; (3) cyclical reinforcing and reviewing training leads to a detailed categorization as well as to a detailed clustering, while keeping the clusters that have already been learned and the network structure that has already been developed stable; and (4) reinforcing-by-link training leads to well-perceived auditory–semantic associations. Our I-GSOM model suggests that it is important to associate auditory information with semantic information during language acquisition. Despite its high level of abstraction, our I-GSOM approach can be interpreted as a biologically-inspired neurocomputational model.

## 1. Introduction

During language acquisition, children receive various kinds of information through their interactions with communication partners and the surrounding environment. In this process, children are presented with information from different channels simultaneously, including auditory information (which can be acquired through auditory feedback), somatosensory information (which can be acquired through tactile feedback from their articulators) and semantic information (which can be abstracted from visual feedback, tactile feedback, olfactory feedback, etc.). Children face the task of acquiring information and organizing it into the appropriate linguistic categories. However, children do not receive explicit language instruction, nor are they able to make inquiries about the structures that they are learning (Gauthier et al., 2007a). Instead, they must discover the linguistic categories of their native language through their interactions with communication partners. This task is further complicated by the fact that they do not know how many categories there are to discover along any particular input dimension (Gauthier et al., 2007a). Language acquisition, for children, is a combination of statistical learning and (social) communicative learning in which they combine pattern detection and computational abilities with special social skills (Kuhl, 2004, 2010). In the present paper, we propose a feasible approach that is able to explain the acquisition of auditory categories, semantic categories and the associations between auditory and semantic information.

From the perspective of modeling, language acquisition can be abstracted as a knowledge acquisition process. Among various kinds of knowledge acquisition algorithms, Kohonen (1982, 1990, 2001, 2013) introduced the idea of self-organizing neural networks, known as the Self-Organizing Map (SOM), which has the ability to project high-dimensional data onto a low-dimensional feature map. Its highly visual nature (especially in the case of two-dimensional feature maps) enables analysts to gain an overview of the underlying category structures of a data set. Ritter and Kohonen (1989) were among those who first applied the SOM algorithm to linguistic tasks. Their research on semantic modeling revealed that SOM has the ability to detect the “logical similarity” between words and group similar words into clusters. In recent years, the topography-preserving ability and the self-organizing ability of the SOM have been increasingly applied to tasks involving the modeling of acquisition, especially in linguistic field, covering many areas including tonal acquisition (Gauthier et al., 2007a,b, 2009), lexical acquisition and development (Li et al., 2004, 2007; Hernandez and Li, 2007), bilingual lexical development (Zhao and Li, 2007, 2008, 2010; Li, 2009; Zinszer and Li, 2010), grammatical acquisition (Li and Zhao, 2009; Zhao and Li, 2009), semantic representation (Zhao et al., 2011), the relation between sensory, and motor states (Kröger et al., 2006a,b), the acquisition of vowel and consonant auditory categories (ger et al., [Bibr B40]), the acquisition and development of articulatory movements (Kröger et al., 2011a,b; Warlaumont et al., 2013), etc. Li and Zhao (2013) provide an excellent review of SOM-based language models. It has been shown that SOMs can be used to model the topographic structure of knowledge and the self-organizing process of knowledge acquisition.

The essential feature of a knowledge acquisition process is the dynamic scalability of knowledge (i.e., both knowledge and the fields it covers continually increase during the process). Although SOMs work well for modeling the topographic structure and the knowledge reorganization of a learning process, they do have limitations in modeling the incremental nature of knowledge growth. The fundamental problem here is the phenomenon of “catastrophic interference” (French, 1999). If a SOM network is trained to acquire 100 words, for example, and then the trained network is applied to train on another 100 new words, the addition of the second set (*or* new knowledge) will disrupt the learning result of the first 100 words (Li et al., 2004). Therefore, SOMs have difficulties in integrating new knowledge into an existing trained network. In other words, the structure of a SOM cannot be extended easily and thus cannot be directly used to model the knowledge acquisition process realistically.

Exploring extendable SOMs in the field of data mining, Alahakoon et al. (2000) proposed an extendable self-organizing neural network called the Growing Self-Organizing Map (GSOM), which allows new nodes to smoothly join the existing network and dynamically extend the size of the network. Its dynamic structure was shown to be very effective for knowledge discovery applications (Alahakoon et al., 2000). In our study, the Growing Self-Organizing Map algorithm is adapted for the modeling of language acquisition.

Due to the fact that auditory information and semantic information are presented to children simultaneously, the associations between these two types of information are specially taken into account in our model. As described in Kröger and his colleagues' language acquisition model (Kröger and Heim, 2011; Kröger et al., 2011a,b), auditory information and semantic information are acquired at two different levels. Therefore, we use two separate maps for modeling the acquisition of auditory information and semantic information, respectively.

Taking the structure of the DevLex model (Li et al., 2004) and connectionist SOM model (Zinszer and Li, 2010) as a point of departure, in this study, we propose an interconnected self-organizing neural network model which consists of an auditory growing map, a semantic growing map, and associative links between the two maps (see section 2.2 for a description of the model). Although the overall structure of our model is similar to that of the DevLex and the connectionist SOM, there are three main differences. (1) While the previous models simulate the phonological–semantic interface, our approach directly simulates the phonetic–semantic interface. Also, in DevLex, the connectionist SOM, and DevLex-II (Li et al., 2007; Li and Zhao, 2013), it is reasonable to link phonological representations with semantic representations since those models aim at modeling later phases of language acquisition such as vocabulary spurts. In our I-GSOM approach, in contrast, we attempt to model language acquisition in early phases, such as the babbling and imitation stages, in which no phonological representations exist. Phonological representations are generally language specific, so they are a result of early language learning. Thus, the development or emergence of phonological representations relies on the early acquisition of phonetic and semantic categories (Kröger et al., 2011a,b; Eckers and Kröger, 2012). (2) In each growing map, GSOM is used instead of GMAP or SOM to better capture the growing nature of knowledge acquisition. GSOM has a simpler structure and great extendibility, so it is more suitable for complex linguistic modeling tasks. Based on the GSOM training procedure developed by Alahakoon et al. (2000), a cyclical reinforcing and reviewing training procedure is introduced to capture communicative effects during early language acquisition. (3) Novel associative link definitions and weight update rules for associative links are introduced, including a reinforcing-by-link training procedure and a link-forgetting procedure, which capture the communicative effects and the feature of forgetting in auditory–semantic-information linking.

## 2. Materials and methods

### 2.1. Training data

#### 2.1.1. Audio data

To simplify the tasks of audio post-processing and neural representations, the audio recording was conducted on the level of single syllable, with three syllable types, *V, CV* and *CCV* (*V* stands for vowels, *C* stands for consonants). Phonemes comprising our recording syllables were selected from Standard German: five vowels [i], [e], [a], [o], [u]; six plosives [b], [p], [d], [t], [g], [k]; two nasals [m], [n]; and the lateral approximant [l]. When pronouncing isolated vowels in Standard German, a glottal stop [Ɂ] is inserted before the vowel (i.e., [a] is pronounced as [Ɂa]). In total, 70 syllables were listed as our recording scripts.

The voice in the audio recording was provided by a female speaker of Standard German with no speaking or hearing deficits (26 years old). The speaker was asked to produce each syllable three times. Carrier sentences were used during the recording. In total, the audio recording consists of 210 records (70 syllables × 3 realizations). The sampling rate for the recording is 44.1 kHz.

An important characteristic of a speech signal is duration. However, the modeling of the temporal information of a training stimulus is limited in neurocomputational models such as SOM and GSOM: it is only possible to model a temporal succession of training items (training stimuli) and a co-occurring temporal succession of changes of link weights. To overcome this problem, in our approach, the speech signals are converted into neural representations of auditory states (*or* spectrograms) according to the method outlined in Kannampuzha et al. (2011) and Pasley et al. (2012). Calculations were done through a series of processing steps.

The first step is annotation. The time instant of *release* of obstruction (closure or constriction) was marked. For *V* syllables, the *release* position was marked at the release of the glottal stop [Ɂ]; for *CV* syllables, the *release* position was marked at the obstruction release of the *C*; for *CCV* syllables, the *release* position was marked at the obstruction release of the second *C*. Then, the time duration for the part before the *release* position and for the part after the *release* position were calculated for each speech signal. Maximum durations of those two parts were calculated for all 210 speech signals.

The second step is signal alignment and normalization. The first-order derivation was applied to the speech signal acting as a 6 dB/oct high pass filter to filter out F0 interference. Then, all signals were aligned based on their *release* positions and normalized to the same duration length by zero-padding signals with shorter duration length before or after the *release* position to the maximum duration length. Alignment and normalization are crucial processes for calculating our neural representations. Alignment guarantees that, between differently-aligned signals, the same time point carries the same type of acoustic features. Normalization guarantees that each speech signal has the same duration, so that the neural representations of training vectors of each speech signal can be represented in the same length of time (i.e., by the same number of neurons).

The third step is calculation of the spectrogram representations. A 2048-point FFT was conducted on each speech signal, with 256-point Hamming window overlapping every 45 sampling points. Therefore, along the frequency dimension, there are 2048 sampling points, which guarantees high frequency resolutions. Along the time dimension, the window length is 256/44, 100 ≈ 5.8 ms, which guarantees the retrieval of wide-band spectrum information. The time resolution is as high as 45/44, 100 ≈ 1 ms/sample. The amplitude information was then converted to dB representations by Equation (1) with upper boundary 100 dB and lower boundary 60 dB, where 2 × 10^−5^ is a reference amplitude set according to the lowest perceivable amplitude by humans (Reetz and Jongman, 2011). Linear normalization was then performed on each signal locally to normalize the amplitude values of signal representation into the interval between 0 and 1. This is done in order to guarantee that the amplitude information of each signal can occupy the full neural activation space.

(1) $$dB=20\times \log\text{​}\left(\frac{\left\|\text{amplitude}\right\|}{2\times 10^{-5}}\right)$$

**Figure 1:**
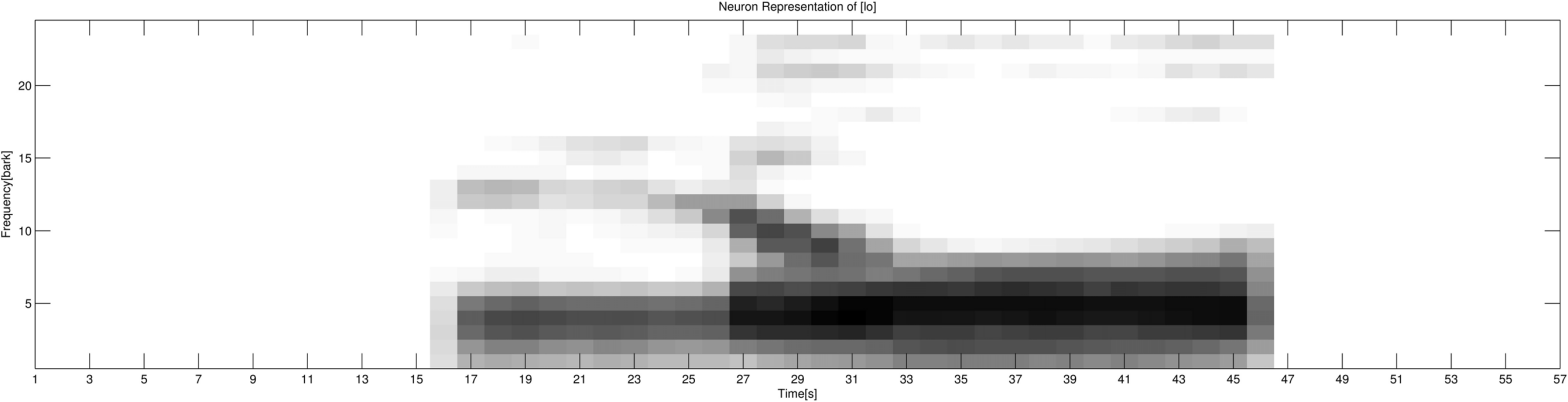
**The auditory neural representation of the acoustic signal of the syllable [lo]**. The frequency dimension is represented by 24 neurons, and the time dimension is represented by 57 neurons. The gray-scale value represents the activation of each specific neuron: 0 or white stands for no activation; 1 or black stands for full activation.

The fourth step is conversion of the spectrogram representations into neural representations. Along the frequency dimension, frequency information was converted into 24-Bark scale representations. Then, each Bark neural group was calculated on the basis of the mean amplitude of all frequency bands occurring within that specific Bark group. Along the time dimension, time information was calculated on the basis of the mean of every 10 samples. As a result, the frequency dimension is represented by 24 neurons (representing the 24 Bark groups) and the time dimension is represented by 57 neurons (each representing a time window of approximately 10.2 ms). In total, each speech signal is represented by 24 × 57 = 1368 neurons. A linear normalization was then performed locally on the neural representation of each signal, so that the normalized amplitude of each signal would fit into the interval between 0 and 1. Thus, the degree of neural activation is represented by the amplitude (gray-scale value) of each neural representation (0 or white stands for no activation; 1 or black stands for full activation). An example of the auditory neural representation of the syllable [lo] is given in Figure 1. As shown in Figure 1, along the frequency dimension, the formant information of segments [l] and [o] as well as a clear formant transition between the two segments can be observed; along the time dimension, the duration information of each segment and the whole syllable are clearly presented.

#### 2.1.2. Semantic data

A Standard German children's books corpus (Kröger et al., 2011a) was used as the basis for generating our semantic data set. The corpus comprises transcriptions of 40 books targeted to children from age 1 to 6. In total, 6513 sentences and 70,512 words are transcribed in the corpus. Morphologically distinct forms of the same word are treated as separate words (e.g., “*Kind*” meaning “child” and “*Kinder*” meaning “children,” are treated as two different words). The corpus therefore consists of 8217 different words, which is assumed to approximately represent a 6-years-old child's mental lexicon, as reported in Anglin et al. (1993). In this study, only nouns were used for our experiments, and only the 70 most frequent nouns were chosen in order to fit into the 70-syllable list. The 10 most frequent nouns are listed in the left columns of Table 1.

**Table 1:** **The 10 most frequent nouns (left) and semantic features (right) in the 70-word data set**.

	**Nouns**		**Semantic features**	
**Rank**	**German words**	**English translation**	**German descriptions**	**English translation**
1	Mama	Mom	Hat zwei Augen	Has two eyes
2	Bär	Bear	Hat eine Nase	Has a nose
3	Papa	Dad	Hat einen Kopf	Has a head
4	Mond	Moon	Hat zwei Beine	Has two legs
5	Kinder	Children	Ist ein Gegenstand	Is an object
6	Katze	Cat	Ist ein Tier	Is an animal
7	Frau	Wife	Hat eine Haut	Has a skin
8	Bett	Bed	Hat zwei Arme	Has two arms
9	Mädchen	Girl	Hat einen Mund	Has a mouth
10	Wasser	Water	Es gibt verschiedene Arten	There are different types

Two native speakers of Standard German (undergraduate students at RWTH Aachen University) developed a list of semantic features for the corpus using a simple brain storming procedure. Thus, 470 features in total were developed for the 70 nouns. To reduce the dimensions of the training vector, we dropped features that only occur once, while keeping all words distinguishable by their semantic features. In other words, a feature was dropped if it occurred only once, but if the deletion would have resulted in two words having the same vector value, the feature was then retained. Finally, 361 features were kept (the 10 most frequent features are listed in the right columns of Table 1). Therefore, each word in our semantic data set is represented by the set of features we kept. Binary coding is used to represent the semantic features of each word. Thus, for each word, among the set of binary features we kept, “1” is used to mark the features of the word, and “0” is used to mark features not belonging to this word.

#### 2.1.3. Acoustic–semantic (sound–meaning) pairs

Since auditory information (the sounds of a word) is associated with semantic information (the meaning of a word), during the training process, audio data and semantic data should be presented simultaneously to the network. However, due to our simplification during audio recording, our acoustic representations are not directly correlated with the word form representations in the semantic data set, so associations between our audio data and our semantic data needed to be developed. The arbitrary associations between the phonetic representations and semantic features of words were assigned by creating a model language (Kröger et al., 2011c). Thus, acoustic–semantic (*or* sound–meaning) pairs were built.

In total, there are audio data for 70 syllables with 3 realizations each and semantic data for 70 nouns. A basic rule we applied was pronunciation similarity. For example, the audio syllable [ma] was chosen as the acoustic realization of the word “Mama,” and acoustic–semantic pair [ma]—“Mama” was built. Inevitably, there were exceptions, in that some words could not be matched to acoustic realizations with similar pronunciations. In that case, those words were manually paired with the remaining audio syllables, based on the fact that the association between the phonetic and semantic values of a word is mainly arbitrary and differs from language to language. While three realizations of an audio syllable are treated as three different acoustic signals, they share the same acoustic–semantic pair relation (i.e., the second and third acoustic realization of the syllable [ma] are also paired to the word form “Mama”). In total, 210 acoustic–semantic pairs were built. Those acoustic–semantic pairs represented by their corresponding acoustic and semantic feature were used as the training data for our experiments.

### 2.2. Description of the model

**Figure 2:**
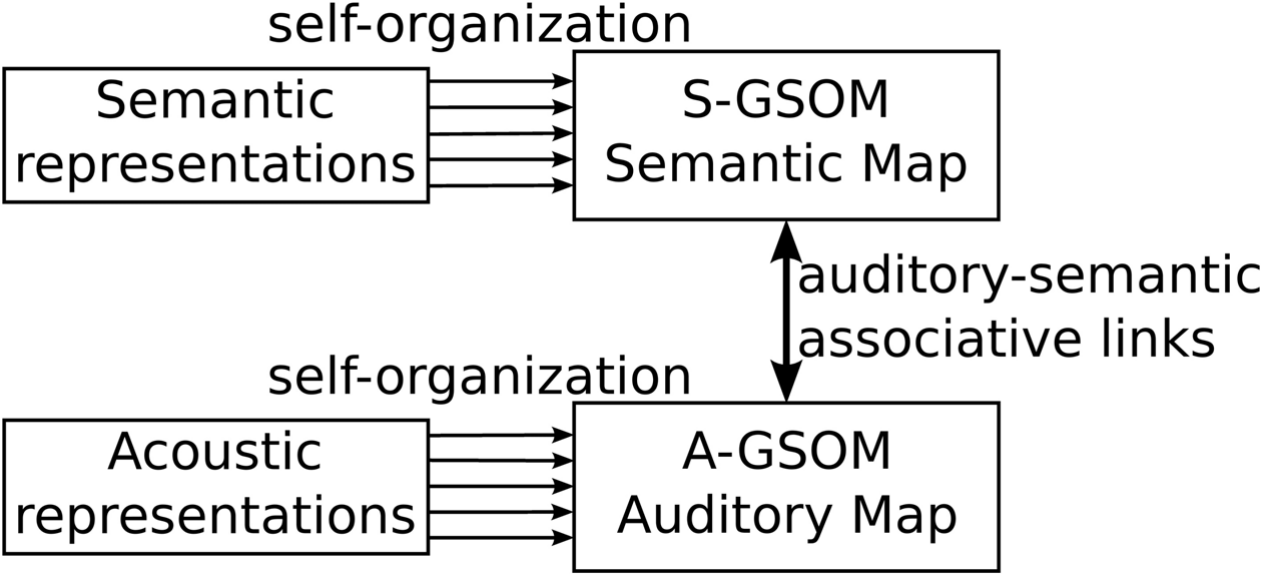
**The Interconnected Growing Self-Organizing Maps model**.

The Interconnected Growing Self-Organizing Map (I-GSOM) consists of two growing maps connected by associative links between them (see Figure 2). An auditory growing map is used to process auditory information, and a semantic growing map is used to process semantic information. Each growing map is defined and trained based on the same GSOM algorithm, but the biological structures they represent are different. The associative links between the two maps are defined as bidirectional links that associate the activations in the two maps. Links are only built and updated between the winner neuron in the auditory map and the winner neuron in the semantic map for a given acoustic–semantic pair (see section 2.4 for detailed training algorithms for associative links).

### 2.3. Structure of the growing self-organizing map

**Figure 3:**
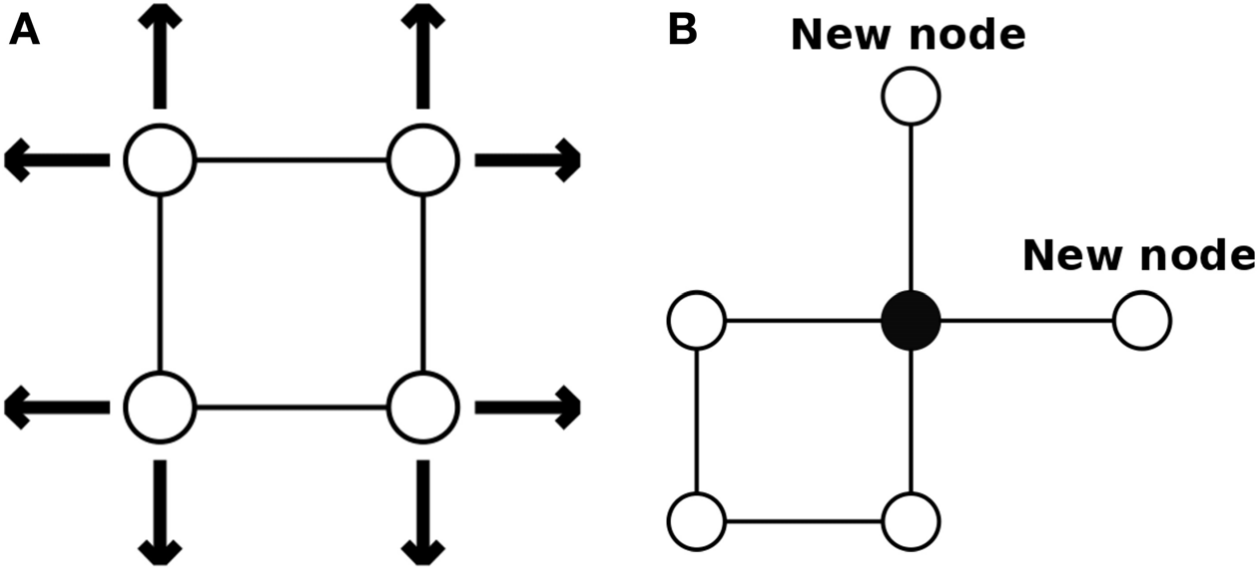
**The initial structure of a GSOM**. **(A)** The network can be expanded in any direction at the beginning. **(B)** New nodes can expand the network at boundary nodes (Alahakoon et al., 2000).

Compared with traditional SOMs, the structure of Growing Self-Organizing Map (GSOM) is simpler. Instead of having a rectangular map of a predetermined size, the network of GSOM does not have a fixed size or shape. Starting with four initial nodes, new nodes can grow at boundary nodes and smoothly join the existing network (see Figure 3B). Thus, the network can be expanded dynamically in any direction outwards depending on the new growing nodes (see Figure 3A).

Two factors, the accumulative error (*E*_acc_) and growth threshold (*T*_grow_), are introduced into GSOM. The error value is calculated on the basis of the Euclidean distance between an input vector and the weight vector of the best matching unit (BMU). Thus, each BMU node has an error value as an additional characteristic parameter, and its value is accumulated throughout the training process. When the *E*_acc_ value of a BMU exceeds *T*_grow_, the corresponding Voronoi region (Okabe et al., 2009) is said to be underrepresented, and new nodes are then introduced into the network. A high *T*_grow_ value will result in a map with fewer nodes, while a low *T*_grow_ value will produce a map with more nodes (Alahakoon et al., 2000).

### 2.4. The basic growing training procedure

Training data (i.e., a set of acoustic–semantic pairs represented by feature vectors) are treated as input tokens for the network training process. The basic growing training process of our I-GSOM model contains two phases described in the following two sections. The pseudo algorithm of the basic growing training process is demonstrated in Algorithm 1.

**Algorithm 1:** **The basic growing training procedure**.

1: **procedure** Initializing Phase
2: initialize the growth threshold (*T*_*grow*_)
3: **for** each of the 4 initial nodes in *AMap* and in *SMap* **do**
4: initialize the accumulated error (*E*_*acc*_) of the node to **0**
5: randomly initialize the weight vector of the node
6: **end for**
7: **end procedure**

8: **procedure** Growing Phase
9: **for** each training token **do**
10: initialize the neighborhood size and learning rate
11: present the audio training data to *AMap* and the semantic training data to *SMap*
12: identify the BMU in *AMap* and the BMU in *SMap*
13: **for** the BMU in each map **do**
14: **if** *E*_*acc*_ > *T*_*grow*_ **and** the BMU is a boundary node **then**
15: grow new nodes
16: initialize the weight vector of each new node
17: **else**
18: **while** neighborhood size ≥ 1 **do**
19: **if** *E*_*acc*_ ≤ *T*_*grow*_ **then**
20: update the weight vector of the BMU and its neighbors
21: calculate the error value and accumulate it to *E*_*acc*_ of the BMU
22: **if** an associative link exist between BMU in *AMap* and BMU in *SMap* **then**
23: update the weight of the associative link
24: **else**
25: build an associative link between the two BMUs
26: initialize the weight of the associative link
27: **end if**
28: **else if** the BMU is *not* a boundary node **then**
29: do error distribution
30: **end if**
31: reduce the neighborhood size and learning rate
32: **end while**
33: **end if**
34: **end for**
35: **end for**
36: **end procedure**

#### 2.4.1. The initializing phase

At the beginning, four neurons are initialized in the auditory map, and four are initialized in the semantic map. The feature values of their weight vectors are assigned randomly within the interval 0–1, and their *E*_acc_ values are initialized to 0. In contrast to the approach of Alahakoon et al. (2000), in our approach, *T*_grow_ is set arbitrarily to fit our experimental requirements.

After initialization, all our starting nodes are boundary nodes and thus free to grow in any direction outwards. This results in great flexibility in terms of network growth.

#### 2.4.2. The growing phase

- Input tokens are presented to the network one by one sequentially. Audio data are presented to the auditory map for training, and simultaneously, the corresponding semantic data are presented to the semantic map for training. Each token is trained several times before moving to the next. This approach is consistent with the gradual learning process in natural language acquisition: parents often teach their children just one word at a time and repeat it several times.
- In GSOMs, the weight updating and network reorganizing processes are performed locally. Therefore, the learning rate and neighborhood size are initialized to their initial value with each new input token. The learning rate (*R*_learn_) update rule is defined as a function of the total number of nodes in the network, i.e., *R*_learn_(*t* + 1) = *α* × *ϕ*(*n*) × *R*_learn_(*t*), where α is the reduction factor of the learning rate with 0 < *α* < 1; *ϕ*(*n*) = 1 − *Q*/*n*(*t*) is a function of the total number of current nodes in the network; *Q* is a constant set to 3.8 since the starting number of nodes is four (Alahakoon et al., 2000); and *n*(*t*) is the number of nodes in the network at time *t*. Since the auditory map may have a different size compared with the semantic map during the growing process, the decreasing rates of the learning rate are not exactly the same for those two maps.
- The distances between weight vectors and the vectors of input training tokens are calculated using the Euclidean distance measure, and the best matching unit (BMU) with minimum distance is detected simultaneously within the auditory map and the semantic map for the current training token.
- In each map, if the *E*_acc_ value of a BMU exceeds *T*_grow_ and this BMU is a boundary node, then new nodes grow at all free directly adjacent positions. The weight vectors of new nodes are initialized with regard to the weight vectors of the relevant BMU and its direct neighbors. (The process performed in Step d is called *weight distribution*.)
- In each map, if the *E*_acc_ value of a BMU does not exceed *T*_grow_, then a weight update is applied to the BMU and its neighbors within the neighborhood. Gaussian distribution is chosen as a part of the neighborhood function that can be represented by *h*(*t*) = exp (−*d*^2^_*ix*_/2*σ*(*t*)^2^), where *d*_*ix*_ represents the Euclidean distance between the weight vector ω_*i*_ in the BMU and the training vector *x* in the training token; σ(*t*) represents the current neighborhood size. The value of *σ*(*t*) is calculated by *σ*(*t*) = *β* × *σ*(*t* − 1), where *β* is the reduction factor of the neighborhood size with 0 < *β* < 1; *σ*(*t* − 1) represents the neighborhood size in the previous state. The weight update function can be expressed as in Equation (2). The Euclidean distance between this BMU vector and the training vector is accumulated as the *E*_acc_ value of the BMU.
  (2) $$\omega_{i}(t+1)=\omega_{i}(t)+R_{\text{learn}}(t)\times h(t)\times \left(x(t)-\omega_{i}(t)\right),i\in N$$
  If an associative link already exists between the BMU of the auditory map and the BMU of the semantic map, the weight of the associative link is then updated using the weight update rule defined in Equation (3), where *L* is a constant set to 0.1 in our experiment. If the link does not exist, a new associative link is then established between the two BMUs, and its weight is initialized to 0.1.
  (3) $$\omega_{\text{link}}(t+1)=\omega_{\text{link}}(t)+L$$
- In each map, if the *E*_acc_ value of a BMU exceeds *T*_grow_ but this BMU is not a boundary node, then error distribution is performed. Thus, the error value of this BMU is reduced to *T*_grow_/2, and the error values of its immediate neighbors increase by *γ* × *T*_grow_, where γ is a factor of *error distribution* with 0 < *γ* < 1. (The process performed in Step f is called *error distribution*).
- Several iterations are done for the current training token (Steps c to f are repeated several times). The learning rate and neighborhood size decrease at each iteration. The iteration process stops when neighborhood size reduces to unity.
- Finally, the next training token is processed by repeating Steps b to g until all training tokens have been presented.

### 2.5. The checking process

The checking process does not change the network. It consists of two procedures (the pseudo algorithm of the checking process is demonstrated in Algorithm 2). First, it is performed to check whether the trained network has learned a good representation of the categories represented within the training data. This is done by identifying the winner positions in the trained auditory map and semantic map for each token and can be considered as a calibration phase if known data are used (Alahakoon et al., 2000). The closeness of a token to each neuron in the network is measured by Euclidean distance.

**Algorithm 2:** **The checking process**.

1: **procedure** Check Map Accuracy
2: present checking set to the trained *AMap* and *SMap*
3: identify the BMU in *AMap* and the BMU in *SMap*
4: **end procedure**

5: **procedure** Check Link Accuracy
6: identify the winner link for each solid node in *AMap*
7: **if** the winner link connects to no word representations in *SMap* **then**
8: mark the link as *incorrect*
9: **else if** the connected representation is *wrong* **then**
10: mark the link as *incorrect*
11: **else**
12: mark the link as *correct*
13: **end if**
14: **end procedure**

Second, the accuracy of each associative link is checked. In this study, presently, only the perceiving path (originating in the auditory map and ending in the semantic map) is checked during the checking process. Thus, for each winner in the auditory map, among all associative links originating from that node, the one with the maximum weight is chosen as the winner link of the winner neuron. If the winner link connects to no word representations in the semantic map, the link is marked as *incorrect*; if the winner link connects to a word representation in the semantic map, but that word is not represented by the appropriate audio data, the link is marked as *incorrect*; if the winner link connects to a word representation in the semantic map, and that word is represented by the linked audio, the link is marked as *correct*. The link accuracy can be calculated as the ratio of the number of correct links to the total number of links.

### 2.6. Cyclical reinforcing and reviewing training

During language learning process, children cannot learn everything (i.e., all auditory and semantic categories represented by the training tokens) at once, so imperfections in clustering are inevitable. During language acquisition, errors may represent a current inability to distinguish between words with different sounds or different meanings. Reflected in the network, this fact is represented by those neurons that are found to represent many audio data sequences or words in the checking process. This is comparable to the following real-world learning situation: when parents teach their child, if they find that their child always confuses the same sounds or meanings, parents will repeat those stimuli and reinforce the differences between them in order to help their child learn the difference. During the reinforcement process, some learned sound–meaning pairs would also arise and get enforced in the communication between the child and parents. Therefore, not only the confused words, but also some learned words should be included for further training. The descriptions above involve communicative learning. Since social communication is essential for early language acquisition (Doupe and Kuhl, 1999; Kuhl, 2003; Kuhl et al., 2005), the modeling of communicative effects is important in our model.

When the growing phase is complete and the checking process has been performed, the trained network may end up with some “unsolved” edge nodes, which means that some nodes may represent the characteristics of many tokens comprising different auditory or semantic categories. (These nodes are called “high-density” nodes.) In order to resolve the high-density nodes, a series of reinforcing and reviewing training can be performed on both maps.

For the reinforcing phase, the starting point is the trained network after completing the growing phase, and the training process is similar to that of the growing phase. Training data consist of those acoustic–semantic pairs whose auditory information or semantic information is represented in the high-density nodes, found in the checking process. The initial learning rate is increased to give more weight to the input token, and the *T*_grow_ value is decreased to stimulate the network growth at high-density nodes.

For the reviewing phase, the starting point is the trained network after completing the reinforcing phase, and the training process is similar to that of the growing phase. Compared with the reinforcing phase, more tokens from the training set are used in the reviewing process to simulate the reoccurrence of knowledge that has already been acquired. The initial learning rate and the *T*_grow_ value are set to the same level as in the growing phase.

A reinforcing phase followed by a reviewing phase together form a combined training process of reinforcing and reviewing training. According to our experimental requirements, this combined training process repeats in a cyclical (*or* iterative) way several times until the average number of words represented by a neuron reaches a minimum in both the auditory map and the semantic map (see the left y-axis of Figure 7 in section 3.1). The pseudo algorithm of the cyclical reinforcing and reviewing training process is demonstrated in Algorithm 3.

**Algorithm 3:** **Cyclical reinforcing and reviewing training**.

1: **procedure** Reinforcing Phase
2: perform the checking process
3: select tokens for the reinforcing training
4: assign a smaller *T*_*grow*_ and bigger initial *R*_*learn*_
5: do reinforcing training
6: **end procedure**

7: **procedure** Reviewing Phase
8: select tokens for reviewing training
9: assign *T*_*grow*_ and initial *R*_*learn*_ to the same value as in the growing phase
10: do reviewing training
11: **end procedure**

In this study particularly, the entire training set is used in the reviewing phase. In other words, the reviewing phase in this study is almost the same as the growing phase. The function of the reviewing phase here is to consolidate the knowledge that has already been acquired.

### 2.7. Reinforcing-by-link training

As mentioned above, during the learning process, imperfections in sounds–meaning linking are inevitable. During language acquisition, the errors may represent a current inability to determine the correct meaning of a speech signal. Reflected in the network, this fact is represented by those incorrect links from the auditory map to the semantic map. Therefore, as mentioned in the description of the reinforcing phase in section 2.6, reinforcement is needed to help the network (*or* in the real world, the child) to learn the correct associations between the auditory information and semantic information.

The reinforcing-by-link training does not correct the incorrect links directly. In fact, this is done in a more realistic way: in the real world, parents would repeat those misunderstood sound–meaning pairs and reinforce the relations between them in order to help the child learn the correct associations. Thus, the reinforcing-by-link phase is not introduced to the training process from the beginning, since well-developed auditory and semantic maps are required in order to function as the basis for the process. Therefore, the reinforcing-by-link phase is introduced at a middle stage in our training procedure and is applied before each reinforcing and reviewing cycle. The starting point for this training cycle is the trained network from the previous reviewing phase. The training data consist of those acoustic–semantic pairs whose auditory information or semantic information is represented in the incorrect links from the auditory map to the semantic map. As in the reinforcing phase, the initial learning rate is increased to give more weight to the input token simulating the reinforcement effect, and the *T*_grow_ value is decreased to stimulate the network growth at high-density nodes. In this study, experiments performed with and without the reinforcing-by-link phase were conducted, in order to explore the effect of our reinforcing-by-link training procedure. The pseudo algorithm of the reinforcing-by-link training process is demonstrated in Algorithm 4.

**Algorithm 4:** **The reinforcing-by-link training**.

1: **procedure**
2: perform the checking process
3: select tokens for the reinforcing-by-link training
4: assign a smaller *T*_*grow*_ and bigger initial *R*_*learn*_
5: do reinforcing-by-link training
6: **end procedure**

### 2.8. The link-forgetting procedure

From the simple weight update rule for associative links as represented in Equation (3), we can see that the weight will keep increasing and lead to a constant enhancement of all auditory–semantic links during the training process. However, this does not correspond to natural or real-world mechanisms of how knowledge enters long-term memory. In addition, not everything can be retained in the long-term memory and remembering is a highly selective process. In fact, remembering can cause forgetting (Anderson et al., 1994). The recalling of a remembered item will increase the likelihood that it will be recallable again at a later time, but items that are associated to the same cue or cues as another item may be put in greater jeopardy of being forgotten (Anderson et al., 1994). Moreover, by modeling the language learning process, Barrett and Zollman (2009) showed that forgetting is beneficial for evolving an optimal signaling language. Therefore, while remembering is modeled by the weight update rule represented in Equation (3), forgetting should also be modeled in our approach as an indispensable part of the real-world learning process. In addition, if no forgetting procedure is applied to associative links, our I-GSOM network can run into problems. Since both maps grow outwards from their four initial nodes, the associative links trained earlier will gain too much weight and can easily overpower other links. However, since both networks continually grow and reorganize during the training process, the network structure, as well as auditory and semantic clusters, remains in a dynamic changing process. Therefore, if those links that were trained earlier have very strong weights, the corresponding linked neurons in the auditory map and the semantic map have little possibility of representing paired acoustic–semantic features. Based on these considerations, we introduced a link-forgetting procedure into our training process.

Howe and Courage (1997) reported that over a 3-month delay, 15-month-olds evidenced more forgetting than 18-month-olds, and 12-month-olds evidenced more forgetting than 15-month-olds. Based on their findings, we also assume that younger children may have greater forgetting rates. We interpret this result as indicating that younger children may have a less developed brain structure (network structure) and therefore have less capacity for remembering the knowledge they have already acquired. Based on this idea, a link-forgetting rate, defined as a function of the number of all possible links between the auditory map and the semantic map, is introduced into our model. The number of all possible links (*N*_link_) is calculated by the multiplication of the total number of nodes in the auditory map and the semantic map. Therefore, the initial *N*_link_ equals to 4 × 4 = 16. The link-forgetting rate (Δω_link_) is calculated as in Equation (4), where *N*_link_(*t*) stands for the number of all possible links in the current training state and *N*_link_(*t* − 1) stands for the number of all possible links in the previous training state.

(4) $$\Delta \omega_{\text{link}}=\sqrt{\frac{N_{\text{link}}(t)-N_{\text{link}}(t-1)}{N_{\text{link}}(t)}}$$

The link-forgetting procedure is applied to all existing links in the current trained network when each training phase ends. Thus, it is applied after each growing phase, reinforcing-by-link phase, reinforcing phase, and reviewing phase. The forgetting strategy implemented by Barrett and Zollman (2009) was to reduce the possibility that past partial success would continue to reinforce suboptimal practice. Taking their ideas as a starting point, we defined a preliminary link-forgetting rule as in Equation (5), where ω_link_(*t*) stands for the weight of a link in the current training state and ω_link_(*t* + 1) stands for the weight of the link after the link-forgetting procedure. The pseudo algorithm of the procedure is expressed in Algorithm 5.

(5) $$\omega_{\text{link}}(t+1)=\omega_{\text{link}}(t)\times \left(1-\Delta \omega_{\text{link}}\right)$$

**Algorithm 5:** **The link-forgetting procedure**.

1: **procedure**
2: **for** each link **do**
3: reduce the weight of link by link-forgetting rule
4: **end for**
5: **end procedure**

## 3. Results

**Figure 4:**
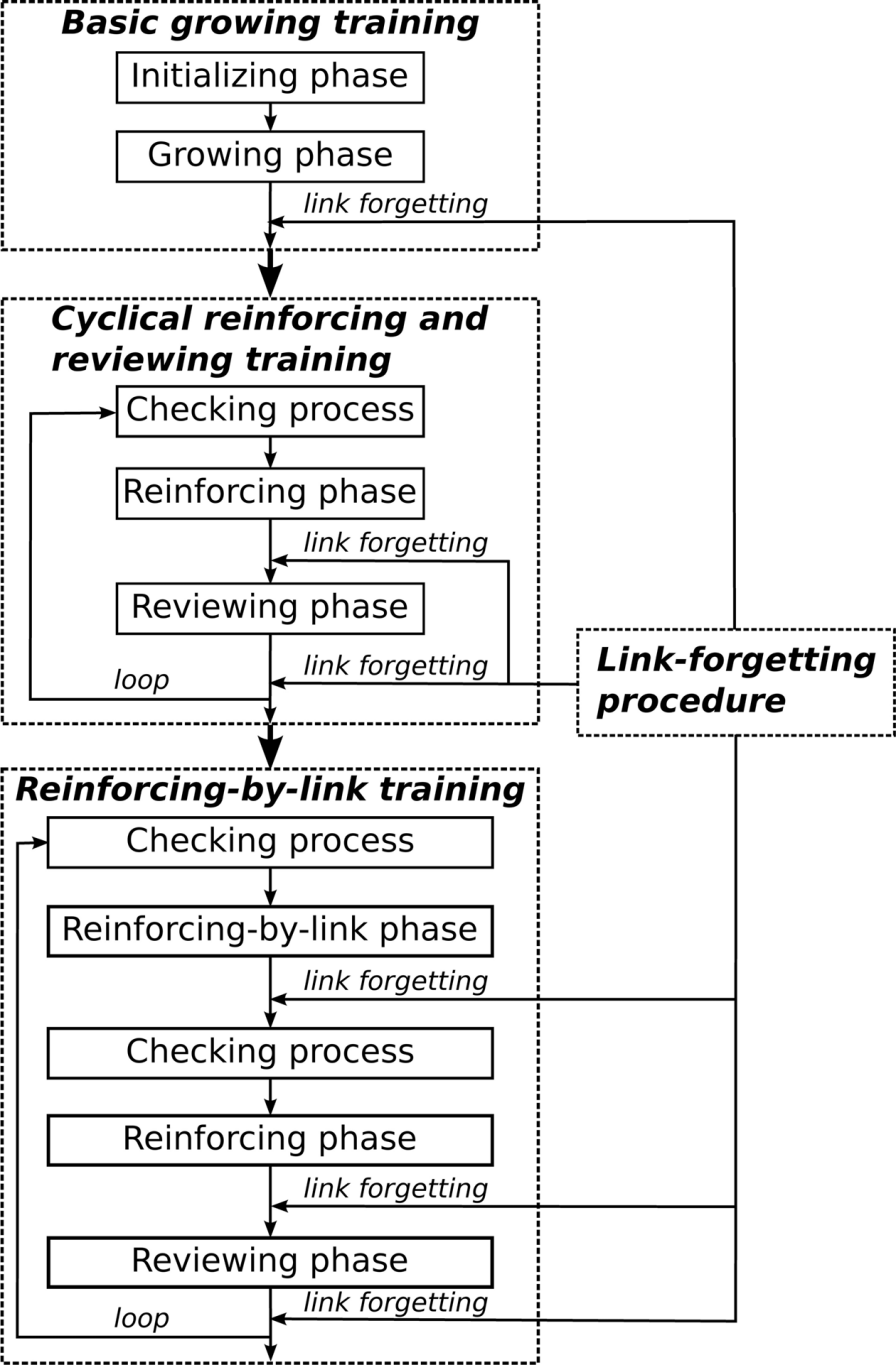
**The order of operations for the training procedures**.

In this section, we present our experiment procedures and experimental results in detail. The order of operations for the training procedures is shown in Figure 4. Three simulations with identical model parameters (listed in Table 2) were performed. From our analysis, all three simulations behave similarly and lead to comparable results. The results reported in sections 3.1 and 3.2 are based on the averages of the three simulations. In section 3.3, we mainly report on, in detail, and discuss the results from our first simulation.

**Table 2:** **Parameters used in the various training phases**.

**Training phases**	**Initial *R*_learn_**	**Initial σ(*t*)**	***T*_grow_**	*α*	*β*	*γ*
Basic growing	0.5	2	2	0.9	0.9	0.5
Reinforcing	0.8	2	1	0.9	0.9	0.5
Reviewing	0.5	2	2	0.9	0.9	0.5
Reinforcing-by-link	0.8	2	1	0.9	0.9	0.5

*Initial *R*_learn_ represents the initial value of the learning rate; Initial σ(t) represents the initial value of the neighborhood size; *T*_grow_ represents the value of the growth threshold; α represents the reduction factor of the learning rate; β represents the reduction factor of the neighborhood size; γ represents the factor of error distribution*.

### 3.1. Fundamental training

The fundamental training is implemented to check the basic performance of our model and build a foundation for later reinforcing-by-link training (since as mentioned in section 2.7, well-developed auditory and semantic maps are required, which form the basis for the reinforcing-by-link training process). The fundamental training consists of a basic growing training phase (see section 2.4) followed by cyclical reinforcing and reviewing training phases (see section 2.6). The training process was divided into 31 steps. Step 1 represents the basic growing training. The following 30 steps represent the steps in the cyclical reinforcing and reviewing training (from Step 2, even numbers represent the reinforcing training steps and odd numbers represent the reviewing training steps). In total, one basic growing training and 15 cycles (30 steps) of reinforcing and reviewing training were performed.

During the reinforcing phase, acoustic–semantic pairs which needed to be reinforced were selected by the following procedure. First, the checking procedure was performed on both the trained auditory map and the trained semantic map after the previous training step. (For the first reinforcing phase, the “previous training step” refers to Step 1; for the following reinforcing phases, the “previous training step” refers to each reviewing phase before the reinforcing phase). Then, if a neuron in the auditory map is found to represent more than four audio data segments, or the average Euclidean distance between a neuron and its represented audio information is greater than 2.5, the corresponding acoustic–semantic pairs which contain those audio data segments are then taken as the training data for the reinforcing phase. At the same time, if a neuron in the semantic map is found to represent more than four words, or the average Euclidean distance between a neuron and its represented words is greater than 2.5, the corresponding acoustic–semantic pairs which contain those words are then taken as the training data for the reinforcing phase. If the auditory information and semantic information of an acoustic–semantic pair are both perceived incorrectly, redundant acoustic–semantic pairs will occur in the training data for the reinforcing phase. In such cases, only one pair was kept. During the reviewing phase, the complete training set was used as the training data.

**Figure 5:**
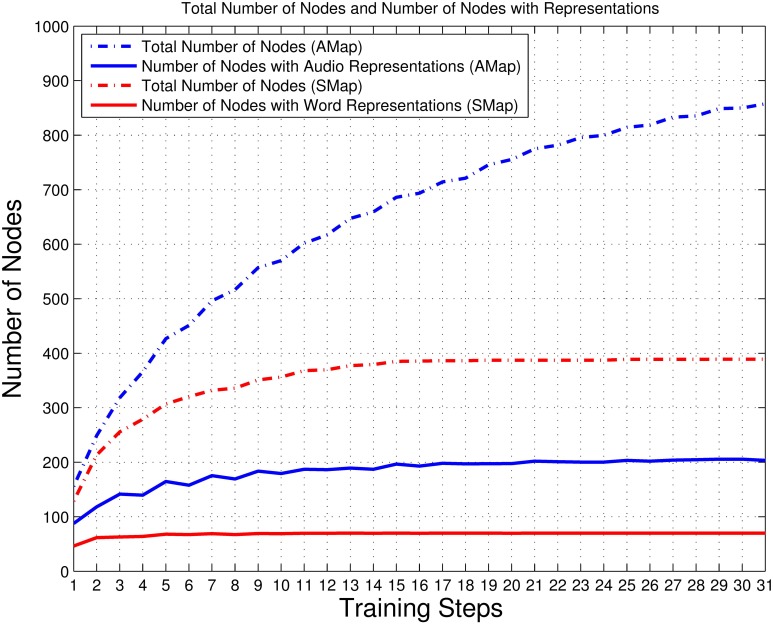
**Total number of nodes and number of nodes with representations in the auditory and semantic maps**. The dotted blue line represents the total number of nodes in the auditory map (AMap), and the solid blue line represents the number of nodes with audio representations in the auditory map. The dotted red line represents the total number of nodes in the semantic map (SMap), and solid red line represents the number of nodes with word representations in the semantic map.

**Figure 6:**
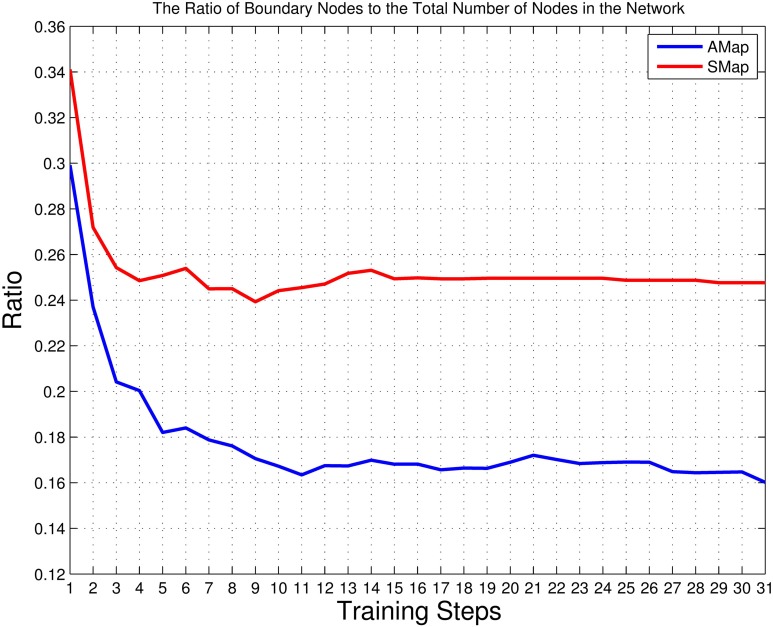
**The ratios of the number of boundary nodes to the total number of nodes in the auditory and semantic maps**. The blue line represents the auditory map (AMap), and the red line represents the semantic map (SMap). This measure reaches a minimum if the shape of the corresponding neural network forms into a circle (the most compact configuration). Thus, this measure reflects the compactness of the shape of a neural network (see Figures 9–13).

With the processing of the training phases, the network keeps growing. Figure 5 shows the growing trends of the total number of nodes and the number of nodes with representations in the auditory map and the semantic map. All curves show a significant increase after the first reinforcing training. The network continually expands, while the increasing rate gradually decreases. In Figure 6, both the ratios of boundary nodes to all nodes for both the audio and the semantic maps decline with the training process and then gradually become stable, which indicates that both maps gradually form a compact network. By comparing the lines representing the auditory and semantic maps in Figures 5, 6, we can conclude that the semantic map achieves a stable state (at Step 15) much faster than the auditory map. This may be due to the fact that the semantic map has fewer unique items to learn than that of the auditory map (70 for the semantic map, 210 for the auditory map). The auditory map ends up with a reasonable size of 857 nodes and a good neuron representation resolution of 203 syllables (96.67% of all syllables represented in the training set are resolved). The semantic map ends up with a reasonable size of 389 nodes and a full neuron representation resolution of 70 words (100%).

**Figure 7:**
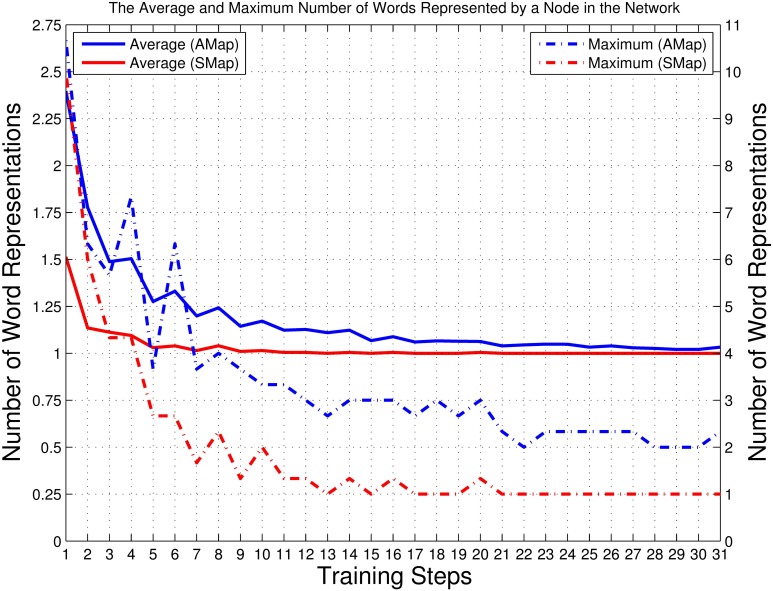
**Left y-axis:** The average number of words represented by a neuron among all those nodes with representations in the auditory and semantic maps. The result was calculated as the ratio of the total number of unique training items (70 for the semantic map, 210 for the auditory map) to the number of nodes with representations (i.e., non-empty, or solid, nodes; see Figures 9–13). The solid blue line represents the auditory map (AMap), and the solid red line represents the semantic map (SMap). **Right y-axis:** The maximum number of audio data sequences represented by a node in the auditory map and the maximum number of words represented by a node in the semantic map. The dotted blue line represents the auditory map (AMap), and the dotted red line represents the semantic map (SMap).

An important aspect of language acquisition is the ability to disambiguate. Thus, in an ideal situation, no neuron should represent more than one neuronal state. As shown in the left y-axis of Figure 7, with the development of the training process, the average number of words represented by a neuron in the semantic map is reduced to 1 and remains stable from Step 11 on; in the auditory map, the number is reduced gradually and approaches 1 in the late stages. The right y-axis of Figure 7 shows the maximum number of words represented by a single neuron in the auditory and semantic maps. Although some fluctuations can be seen during the initial and mid-late stages of the training process, a general declining trend can be found in both lines. Therefore, conclusion can be drawn from above observations that the cyclical reinforcing and reviewing training can help the network resolve high-density nodes, thus can help children to disambiguate the sound and meaning of words in the case of clustered audios or words at one node.

Comparing Figures 6, 7, and observing the structure of the trained network step by step, our results indicate that the cyclical reinforcing and reviewing training can help the network distinguish “unsolved” sound–meaning pairs and build more detailed clusters while keeping the already-acquired network structure stable.

### 3.2. Effects of reinforcing-by-link training

**Figure 8:**
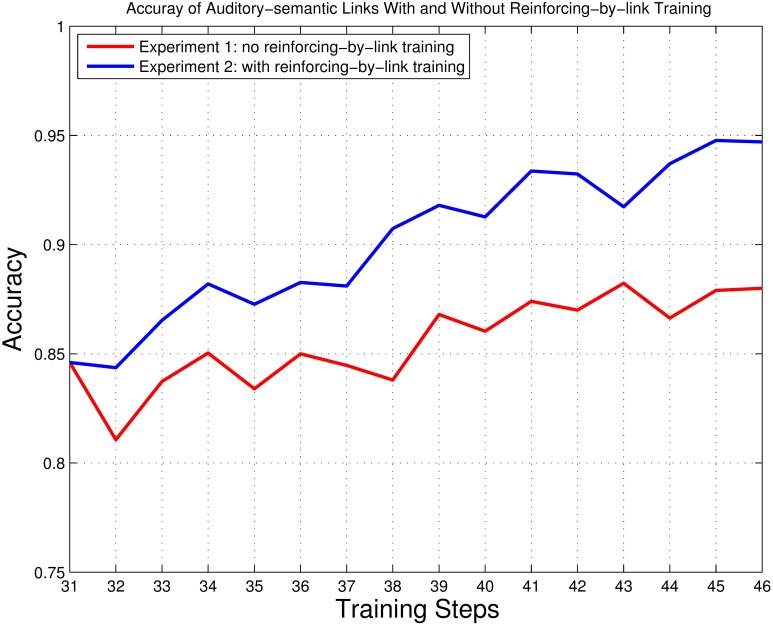
**The accuracy of the associative links for Experiment 1 (no reinforcing-by-link training) and 2 (with reinforcing-by-link training)**. The red line represents Experiment 1, and the blue line represents Experiment 2. Training Step 31 refers to the state of the network as trained through Step 31 of Fundamental training (see section 3.1); Training Steps 32–46 refer to the 15 training cycles preformed in Experiments 1 and 2.

By checking the network trained in Step 1, we found that the accuracy of associative links was poor (see section 2.5 for the accuracy measurement of associative links). Even after a series of further reinforcing and reviewing training phases, as done through Step 31, accuracy still did not reach 90% (see the result of Step 31 in Figure 8). To further investigate the learning of associative links, two series of additional training experiments were performed based on the training results of Step 31. In Experiment 1, we continued training as usual (i.e., without introducing a new reinforcing-by-link training phase). In Experiment 2, we continued training but introduce a new reinforcing-by-link training phase (as described in section 2.7, it is reasonable to introduce the reinforcing-by-link phase at this stage since the auditory and semantic maps are well developed). In both experiments, 15 training cycles were performed. In Experiment 1 (no reinforcing-by-link training), one cycle consists of a reinforcing phase and a reviewing phase. In Experiment 2 (with a reinforcing-by-link training phase), one cycle consists of a reinforcing-by-link phase, a reinforcing phase and a reviewing phase. The trained network resulting from Step 31 was used as the starting point for both experiments. For Experiment 2 (conducted with a reinforcing-by-link training phase), only those links whose weights ranked in the top 20% were taken into consideration during the checking process, since a large amount of links would result in very weak weights, and weak weights have less influences on the selection of winner links. The accuracies of the associative links with and without reinforcing-by-link training were checked in the checking process. The results are presented in Figure 8.

As shown in Figure 8, by introducing the reinforcing-by-link training phase, the accuracy of associative links improves significantly. The associative links end up with 88.00% accuracy in Experiment 1 (no reinforcing-by-link training) and 94.70% accuracy in Experiment 2 (with a reinforcing-by-link training phase). The reinforcing-by-link training phase here models a kind of selection of learning stimuli as it may occur in real-world learning situations. The selected acoustic–semantic pairs are extracted from bad communication results (i.e., from the “misunderstandings” between a child, or in our case, the learning model, and their caretakers; see section 2.7). The results of Experiments 1 and 2 suggest that by presenting the “misunderstood” acoustic–semantic pairs to the network (the child) and reinforcing the sound–meaning associations using reinforcing-by-link training, the network (the child) can develop more accurate associations between paired acoustic and semantic representations.

### 3.3. Network structure analysis

Our analysis of network structure is based on the training result of Experiment 2 (see section 3.2) from our first simulation. After training, the checking procedure was performed simultaneously on the auditory map, the semantic map and the auditory–semantic associative links.

**Figure 9:**
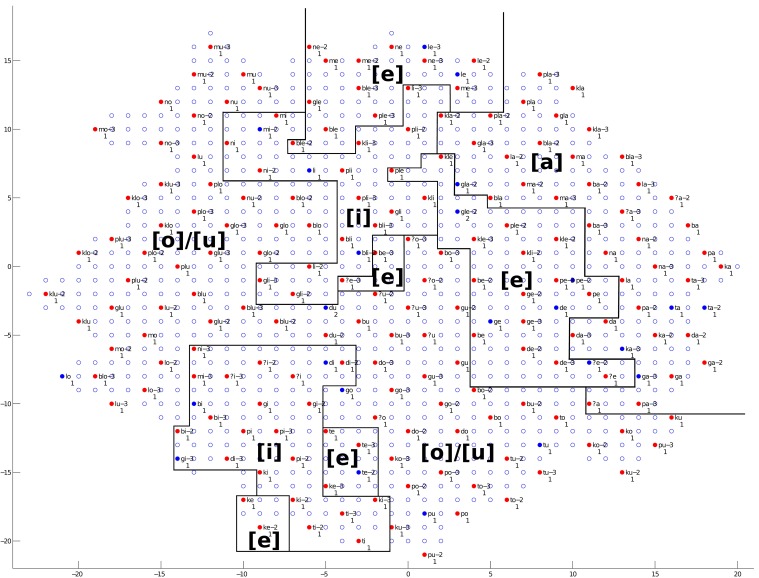
**The trained network structure and checking result of the auditory map for vowel categories**. Clusters of vowel categories are shown. Solid nodes represent those neurons with audio representations; empty nodes represent those neurons with no audio representations. Red nodes represent neurons for which the average Euclidean distance between the neuron and the audio representations it is associated with is smaller than 0.5. Blue nodes represent neurons with an average distance greater than 0.5. The labels next to the nodes give the best-represented audio data sequence for that neuron, and numbers below the nodes represent the number of audio data sequences represented by that neuron. Annotations in the figure represent different clusters, and solid lines represent cluster boundaries.

**Figure 10:**
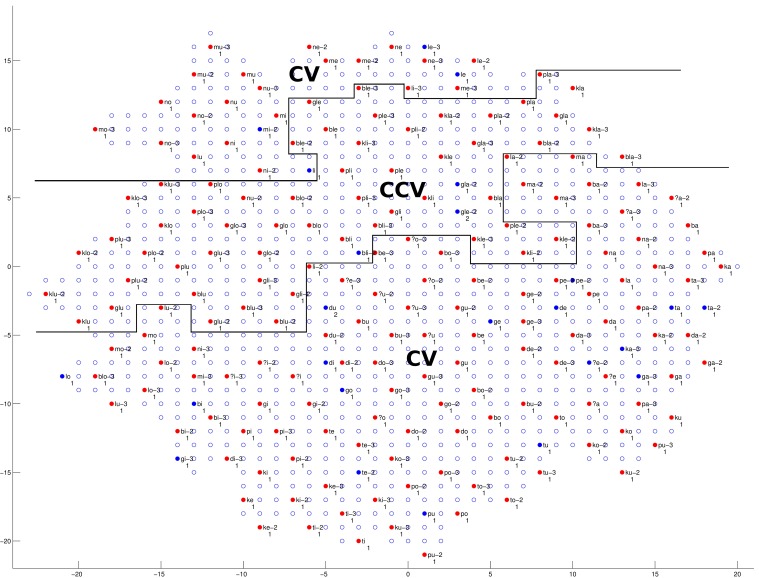
**The trained network structure and checking result of the auditory map for *CV* and *CCV* distinction**. Clusters of *CV* and *CCV* syllables are shown. (See caption of Figure 9 for more details.)

**Figure 11:**
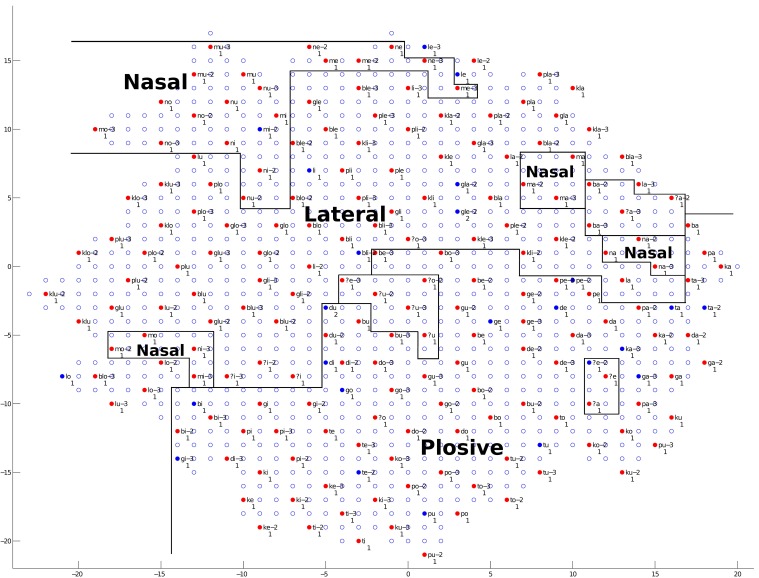
**The trained network structure and checking result of the auditory map for manner of articulation**. Clusters of three different manners of articulation (Nasal, Lateral, and Plosive) are shown. (See the caption of Figure 9 for more details.)

We will first discuss the auditory map. The trained network structure and the checking result of the auditory map are shown in Figures 9–11, demonstrating the clustering result based on vowel categories, *CV*/*CCV* distinctions and manner of articulation, respectively.

As shown in Figure 9, clusters of the five different vowels can be found in various areas. The vowel [a] occupies a stable region in the right and upper right areas of the network. The vowels [i] and [e] occupy relatively stable regions, but there is some interference between the two. The vowels [o] and [u] are mixed together, and no clear cluster boundaries can be found between them. A low–high dimension can be observed in the cluster distribution. The overall trend, with some exceptions, is that low vowels occupy the upper right region, while high vowels occupy the lower left region.

As shown in Figure 10, a clear *CV* and *CCV* distinction can be found, which means the network can successfully distinguish between *CV* syllables and *CCV* syllables. By checking the neural representations of *CV* and *CCV* audio signals (see section 2.1.1 and Figure 1 for an example of a *CV* syllable), we find that *CV* syllables usually have a shorter duration than *CCV* syllables. Therefore, the network (the child) can use duration information as an important cue to distinguish *CV* and *CCV* syllables.

As shown in Figure 11, clusters of the three different manners of articulation (Nasal, Lateral and Plosive) can be observed. By checking the neural representations of audio signals (see section 2.1.1 and Figure 1 for an example of a *CV* syllable), we find that the manner of articulation of consonants can be reflected in the spectrogram information in formant information and formant transitions. Therefore, the network (the child) can make use of spectrogram information to distinguish different manners of articulation among consonants.

Summarizing the findings from Figures 9–11, during the learning (training) process of our model, distinctions between different vowel categories are mainly acquired on the basis of their low–high relations; distinctions between *CV* and *CCV* syllables are mainly acquired on the basis of their duration; and distinctions between different manners of articulation are mainly acquired through the spectrogram information (i.e., formant information and formant transitions) of the syllable. These results, to some degree, explain a possible learning pattern of auditory information in children.

**Figure 12:**
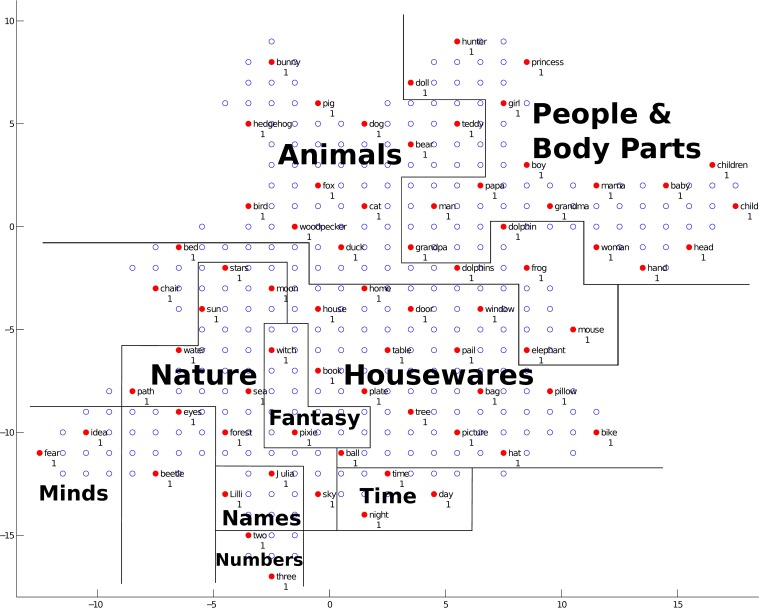
**The trained network structure and checking result of the semantic map**. The clusters of semantic categories are shown. Solid nodes represent neurons with semantic representations, and empty nodes represent neurons with no semantic representations. Red nodes represent neurons for which the average Euclidean distance between the neuron and the word it represents is smaller than 0.5, and blue nodes represent neurons with an average distance greater than 0.5. The labels next to the nodes give the best-represented semantic representation for that neuron, and the numbers below the nodes represent the number of words represented by that neuron. Annotations in the figure represent different clusters, and solid lines represent cluster boundaries.

Next, we will discuss the semantic map. The trained network structure and the checking result of the semantic map are shown in Figure 12. Clusters of different semantic categories can be found in the figure, such as “Animals,” “People and Body Parts,” “Housewares,” “Nature,” “Fantasy,” “Minds,” “Names,” “Numbers,” and “Time.” Within clusters, some fine-grained categorical detail can be seen. For example, in the cluster “Animals,” the grouping of items into the two categories of animals, birds and 4-legged mammals, is reflected in their location, with items from a category clustering together. In the cluster “People and Body Parts,” the items representing men, “grandpa,” “papa,” and “man,” are located close to each other, and the items representing women, “grandma,” “mama,” and “woman,” are located together. In the cluster “Nature,” the items representing heavenly bodies, “sun,” “stars,” and “moon,” are close together, and the earthly items “water,” “sea,” “path,” and “forest” are close together. Between clusters, some clusters with similar meanings are located close to each other. For example, “Animals” is located next to “People and Body Parts” since biologically, humans are a type of animal; “Names,” “Time,” and “Numbers” are adjacent since they are all abstract concepts.

The results for the semantic map demonstrate that our model has the ability to learn the semantic categories of the training data and build semantic clusters. In the mean time, the closeness of different words within one semantic cluster and the closeness of different semantic clusters are generally modeled by our model. At this stage, each solid node in the network represent the semantic features of only one word, and the average Euclidean distances between a node and the word it represents words are all less than 0.5. This means that the semantic map is well developed and the semantic features are well acquired. A possible learning pattern of semantic information for children is demonstrated in Figure 12, in that semantic information is stored with regard to meaning relations (i.e., words with a similar meaning are clustered into one semantic category, and clusters with similar meanings are located close to each other).

**Figure 13:**
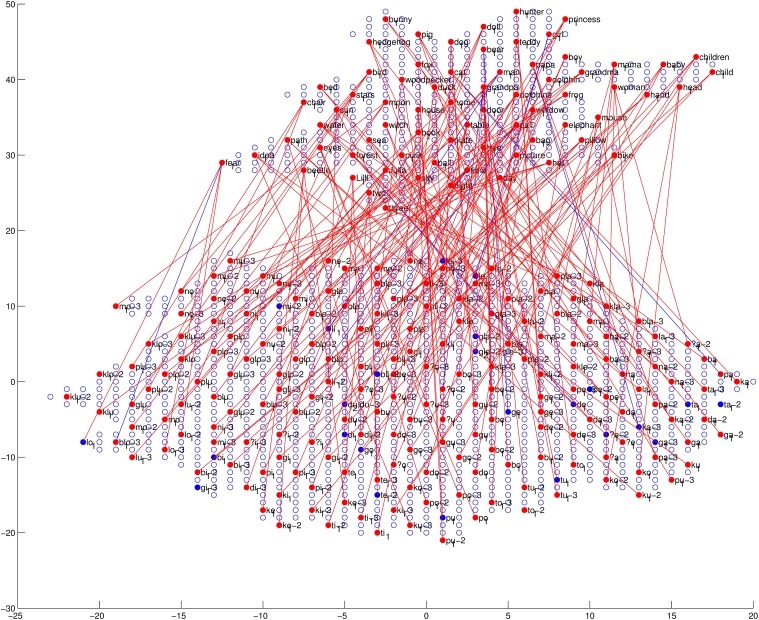
**The trained network structure and checking result of the auditory map, the semantic map and associative links between them**. The red lines represent links with correct auditory–semantic associations, and the blue lines represent those links with incorrect auditory–semantic associations. Among the links, 94.70% are correct. (For detailed information about the auditory and semantic maps, see Figures 9, 12, respectively).

Finally, we will discuss the auditory–semantic associative links. The trained associative links and the accuracy checking result, together with the network structure and checking results of the auditory and semantic maps, are shown in Figure 13. As can be seen in this figure, most activated nodes in the auditory map are linked to the semantic map by associative links. Also, most activated nodes in the semantic map are linked from three nodes (since each word has three corresponding acoustic realizations; see section 2.1.3) in the auditory map by associative links. These results suggest that during the perceiving (checking) process, our trained I-GSOM model has the ability to activate the correct neuron in the auditory map in order to process the corresponding auditory information, then activate the correct associative links to activate the corresponding neuron in the semantic map and find the semantic meaning of the audio input. Therefore by introducing the reinforcing-by-link training phase, the network (the child) can acquire good associations between paired acoustic and semantic representations. The modeling results suggest that during language acquisition, auditory information and semantic information can be linked together and therefore processed and learned simultaneously by children (the model). Thus, the linking relation between auditory information and semantic information is an important aspect of language acquisition.

## 4. Discussion

In this paper, an Interconnected Growing Self-Organizing Maps algorithm (I-GSOM algorithm) is introduced as an approach to modeling the acquisition of auditory information, semantic information and the associations between them. As described in Kröger and his colleagues' language acquisition model (Kröger and Heim, 2011; Kröger et al., 2011a,b), auditory information and semantic information are acquired at two different levels. Therefore, two separate maps (GSOMs) are used to model the acquisition of auditory information and semantic information separately. Taking as a starting point the structure of the DevLex model (Li et al., 2004) and the connectionist SOM model (Zinszer and Li, 2010), associative links are built between the two maps. However, in contrast to the phonological–semantic interface modeled in the DevLex model, the connectionist SOM model and the DevLex-II model (Li et al., 2007; Li and Zhao, 2013), in our model, associative links are built at the phonetic–semantic interface between auditory information and semantic information. It is reasonable to link phonological representation with semantic representation in the previous approaches since those models are used to develop later phases of language acquisition such as vocabulary spurts. In our I-GSOM approach, we attempt to model language acquisition at early phases, such as the babbling and imitation stages, in which no phonological representations are available. Phonological representations are generally language specific, so acquiring them is a result of early language learning. Thus, the development or emergence of phonological representation relies on the early acquisition of phonetic and semantic categories (Kröger et al., 2011a,b; Eckers and Kröger, 2012). The auditory–semantic associated links between the two maps guarantee the perception of associations between paired acoustic and semantic representations, which is very important for early language acquisition.

Also, in contrast to other growing neural network models (Fritzke, 1994, 1995a,b; Bruske and Sommer, 1995; Burzevski and Mohan, 1996; Cheng and Zell, 1999, 2000; Dittenbach et al., 2002a,b; Marsland et al., 2002; Rauber et al., 2002; Li et al., 2004), the GSOM we use has a simpler structure and great extendibility, so it is more suitable for complex linguistic modeling tasks. The self-organizing ability enables it to model the clustering of auditory and semantic categories (i.e., the process of learning acoustic and semantic features), and the dynamic growing structure enables it to model the incremental nature of knowledge growth. By reading in input tokens one at a time and training it for further iterations, the training process of the GSOM simulates the gradual learning process in practice. By introducing the GSOM algorithm based on the accumulative error (*E*_acc_) and growth threshold (*T*_grow_) factors, the network can produce a vivid biological picture of the knowledge acquisition process. The weight distribution passes the weights of the nodes within the neighborhood to the new adding nodes, so that the network structure can remain stable throughout the growing stage. The rules for learning rate adaptation and localized weight update ensure that tokens which enter relatively late can also occupy the appropriate stable regions in the network.

In our I-GSOM algorithm, the auditory and semantic acquisition is modeled using a basic growing training followed by a series of cyclical reinforcing and reviewing training process and a reinforcing-by-link training process, assisted by a link-forgetting procedure. The basic growing training, like most SOM and growing SOM approaches (Li et al., 2004; Kröger et al., 2009b), is a kind of statistical learning process (unsupervised learning). It models language perception based on distributional patterns of sounds and meanings, which provide clues about the phonetic and semantic structure of a language. The reinforcing training steps simulate a situation in which caretakers repeat those words whose sounds or meanings have been misunderstood by the child (the network) and reinforce their specific differences in order to help the child (the network) to correctly distinguish between them. The reviewing training steps simulate the reoccurrence of some knowledge that has already been acquired during the reinforcement learning process. The reinforcing-by-link training steps simulate a situation in which caretakers repeat the “misunderstood” acoustic–semantic pairs and reinforce the correct relations between them, in order to help the child (the network) develop the correct associations. The cyclical reinforcing and reviewing training process, combined with reinforcing-by-link training, forms a kind of communicative learning (semi-supervised learning) process, and it supports language acquisition through a communicative error correction learning mechanism, which is described as the “error processing” module in the DIVA model (Guenther, 2006; Guenther et al., 2006; Guenther and Vladusich, 2012). In our approach, we are among the first to implement this idea in neurocomputational models. Furthermore, remembering and forgetting coexist in real-world learning processes (Anderson et al., 1994). While the weight update rule of GSOM (see Equation 2) models the remembering and forgetting mechanisms for auditory and semantic information, the weight update rule described as in Equation (3) is not capable of modeling the forgetting phenomenon of associative links. Therefore, a link-forgetting procedure is introduced to the associative link learning process. The link-forgetting procedure guarantees the network good accuracy level in auditory–semantic associations.

From our experimental results, we can conclude that our I-GSOM algorithm, in combination with the use of reinforcing and reviewing procedures, demonstrates the ability to learn the acoustic and semantic features in the training data and to build corresponding auditory and semantic clusters in the auditory and semantic maps, respectively. By introducing a reinforcing-by-link training phase, our model demonstrates the ability to associate acoustic features in the auditory map with the semantic features in the semantic map. From the checking results, general vowel clusters, clear *CV*/*CCV* distinctions and clear manner of articulation clusters can be found in the neuron representations of the auditory map; clear semantic clusters can be found in the neuron representations of the semantic map. Thus, we show that cyclical reinforcing and reviewing training is able to help the network distinguish between confused sounds and meanings and build more detailed clusters (i.e., acquire more detailed acoustic as well as semantic features), while keeping the established clusters and the network structure stable. We show that the reinforcing-by-link training phase is able to help the network develop the correct associative links between neurons in the auditory map and neurons in the semantic map (i.e., acquire the correct associations between auditory information and semantic information).

So far, there has been no empirical research on how children acquire auditory information, semantic information and the auditory–semantic associations in the paradigm as we have proposed in this study. In contrast to Li et al. (2004, 2007), our approach currently is not designed to separate training items in a series of increasing lexical items (e.g., 50 words, 100 words, 150 words, … 500 words) as occurs in children's language learning during the first 2 years of life. Thus, our current approach is not yet capable of modeling empirical facts such as the vocabulary spurt occurring around 18 month (which is modeled in Li et al., 2004; Whinney, 2007). In contrast, our approach addresses the problem that children do not have a phonological (i.e., language-specific) speech representation at birth, and it shows how phonological features may emerged. First, we can achieve an ordering of phonetic features at the level of the auditory map based on our syllable repository (see Kröger et al., 2009a). This ordering of language-specific syllable states with respect to phonetic features (i.e., phonetotopy, see Kröger et al., 2009a) is the starting point for building up phonological features. Second, our approach is capable of showing that specific links can be established between neurons (or states) of the auditory and semantic maps. This means that our phonetic ordering of syllables is now linked with language-specific (phonological) distinctiveness, i.e., with different meanings of words (or morphemes). Although there has been no empirical study of the development of phonological features for very young children aged 1–3, we believe that the development of such emerging abilities likely plays an important role in children's early language acquisition.

There are still some drawbacks to our approach, but they can be overcome by further refining the model in future study. Although the I-GSOM provides great flexibility and extensibility, its growing mechanism still has two main problems. (1) New nodes grow at all available positions of the BMU's direct neighbors, which leads to “blind growing” without clear directions. This leads to the problem of redundant nodes. (2) New nodes can only grow at network boundaries and cannot be added to the interior of the network. This limits the expanding process of the network and creates difficulties in modeling real knowledge expansion and visualizing clustering results. Therefore, a further step to improve our model would be to introduce directional growing and interior growing into the I-GSOM algorithm. Related topics have been explored by other researchers: Tai and Hsu (2010, 2012) introduced a “cross insert” growing algorithm, which grows only one new node toward a best-selected direction each time; Ayadi et al. (2007) introduced an Interior and Irregular Boundaries GSOM (2IBGSOM), which makes it possible to add new nodes to the interior of the network. In our future studies, these approaches could be easily integrated into our I-GSOM model.

With respect to the modeling of auditory and semantic acquisition, there are some limitations in our current approach. (1) Acoustic representation displays high variability across speakers. During language acquisition, children must not only build their own acoustic representations, but also learn to deal with acoustic variability in order to be able to understand other speakers. In addition, from the embodied cognition perspective (Barsalou, 2008), semantic representations also differ among individuals. Our I-GSOM approach currently focuses on a back-end knowledge acquisition mechanism, so speaker variability is not taken into consideration at the current stage. Therefore, the front-end speaker normalization is not yet integrated into our approach, and speaker-independent normalization is beyond the scope of this study. In comparison, the DevLex, the DevLex-II and connectionist SOM models are more balanced because they use abstract representations. (2) Words in natural language are formed by one or more syllables. In our approach, as a simplification, only one-syllable words are modeled. Due to the limitations of SOM-based algorithms, training tokens must have the same vector length. Therefore, our I-GSOM approach is not yet capable of dealing with tokens of different vector lengths (i.e., words comprising different numbers of syllables). New data feature representation methods or training algorithms must be developed in the future to address this problem. (3) Language acquisition involves enormous amounts of linguistic stimuli. Both the number of stimuli and the linguistic features those stimuli may have are not precisely modeled in this study. Since our aim in this study was primarily to propose a new modeling perspective, we used a rather small data set consisting of only 70 words (as compared to Li et al., 2004, 2007 in which several hundreds of words were simulated in their models). However, this does not mean that our I-GSOM model is not scalable. Our recent study (Cao et al., 2013), concerning semantic acquisition alone, clearly shows the network scalability of the GSOM algorithm on a relatively large-scale semantic data set (with 1929 training tokens and 724 features in each). Therefore, in term of using our I-GSOM algorithm for large-scale modeling, we expect that the network can form more detailed categories and that some categories can become more compact since more information and more stimuli would be available.

The I-GSOM model we propose is partly connectionist and partly general-computational. On the one hand, the model neurons defined in our approach represent an ensemble of natural cortical neurons, which are spatially and functionally closely connected. Thus, each model neuron may represent, for example, a cortical column (this concept is mainly used in vision, e.g., by Obermayer et al., 1990 and Bednar et al., 2004). On the other hand, the auditory and the semantic maps are connected by simple associative links rather than connectionist activity propagation such as Hebbian learning. In addition, starting from a quite small network size (four nodes), it would also be justified to describe it as a cognitive/computational approach: from the biological perspective, it is known that infants are born with redundant networks (with abundant connections) that go through massive synaptic pruning. Therefore, we state that our I-GSOM model is a biologically-inspired neurocomputational model. Although the I-GSOM is highly abstract, its biological features can be carefully interpreted to a specific degree because it incorporates important neurofunctional principles such as self-organization, associative learning, adaptation, and neural plasticity. Therefore, this kind of simplification on the “microscopic” neurofunctional level allows us to model large-scale and higher-level brain functions and at the same time allows us to model “macroscopic” behavior (e.g., speech learning and speech processing) on the basis of neurofunctional principles.

## Funding

The NSFC Key Project with No. 61233009, and the CASS Innovation Program.

### Conflict of interest statement

The authors declare that the research was conducted in the absence of any commercial or financial relationships that could be construed as a potential conflict of interest.
